# Diagnosing AL and ATTR Amyloid Cardiomyopathy: A Multidisciplinary Approach

**DOI:** 10.3390/jcm13195873

**Published:** 2024-10-01

**Authors:** Fabian aus dem Siepen, Timon Hansen

**Affiliations:** 1 Department of Cardiology, University Hospital Heidelberg, Im Neuenheimer Feld 410, 69120 Heidelberg, Germany; 2 Onkologicum HOPA, 22767 Hamburg, Germany; timon.hansen@hopa.de

**Keywords:** amyloidosis, AL, ATTR, cardiomyopathy, ATTR-CM

## Abstract

Amyloidosis with cardiac involvement is a fatal disease leading to progressive heart failure. The most common forms of amyloidosis with cardiac involvement are light chain (AL) and transthyretin (ATTR) amyloidosis. To allow effective specific treatment for both forms, precise and early diagnosis is important. This review focuses on diagnostic approaches for AL and ATTR amyloidosis with cardiac involvement, including different strategies, the role of imaging and biomarkers and possible pitfalls.

## 1. Introduction

The term amyloidosis labels a group of diseases that lead to extracellular deposition of misfolded proteins that form amyloid fibrils leading to organ dysfunction. Depending on the precursor protein, different organs can be affected.

Considered to be a rare disease in the past, the growing number of patients with cardiac amyloidosis suggests that the disease was underdiagnosed for a long time [1]. This infiltrative cardiomyopathy is a fatal disease leading to progressive heart failure [2]. The most common systemic amyloidosis that lead to cardiac involvement are light chain (AL) and transthyretin (ATTR) amyloidosis [3]. The latter is distinguished in a rare hereditary (variant) subtype and a much more frequent non-hereditary age-dependent subtype. Transthyretin (TTR), mainly produced in the liver, occurs in a tetrameric isoform and physiologically transports thyroxine and retinol. Different mutations in the *TTR* gene can lead to destabilization of the tetramer. After dissociation into monomers, the monomers can form insoluble fibrils, that accumulate as amyloid. Approximately 120 different mutations in the *TTR* gene are potentially amyloidogenic, but even in the absence of an amyloidogenic mutation, with increasing age, an instability of the wild-type protein can occur. The wild-type form (ATTRwt) is mostly seen in older men and is characterized by progressive cardiomyopathy. In addition, soft tissue ligamentous Amyloid deposits lead to stenoses of carpal tunnel, annular ligament or spinal canal [4]. In the variant form (ATTRv), deposits of mutated TTR are found with many potentially involved organs and a TTR-gene mutation-dependent phenotype [5,6]. In AL amyloidosis, misfolded monoclonal light chains, originating from a b-cell clone in the bone marrow, cause multiorgan dysfunction [7] with the heart as most frequently involved organ (75% of cases).

**Figure 1:**
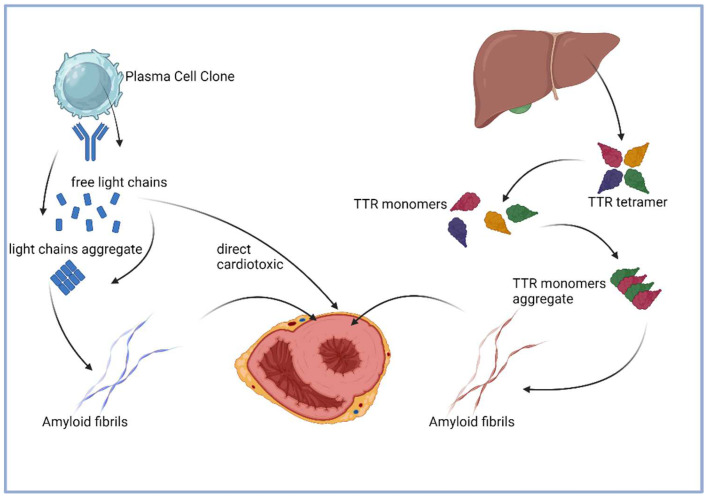
Pathogenesis of AL and ATTR amyloidosis, adapted from Donnelly et al. [8] (illustration created with BioRender).

Organ damage in amyloidosis is mainly caused by the displacement effect of amyloid depositions. Amyloid fibrils accumulate in the interstitial space of the tissue, affecting the physical characteristics of the tissue and impairing the coordination between cells with increasing amounts. Additionally, in AL amyloidosis, circulating free light chains have direct cardiotoxic effects (Figure 1).

Considerable progress was made in the field of therapy over the years and several therapies are available for AL [9,10] and ATTR [11], whereas the type of therapy depends on the amyloid type, involved organ systems and disease stage. Thus, precise and multidisciplinary diagnostics is necessary to enable an appropriate therapy. This review focuses on diagnostic approaches for AL and ATTR amyloidosis with cardiac involvement, including different strategies, the role of imaging and biomarkers and possible pitfalls.

## 2. Clinical Suspicion of Cardiac Amyloidosis and Red Flags

Amyloid infiltration of the myocardium leads to myocardial stiffness, diastolic dysfunction and biventricular systolic dysfunction in the advanced stages of the disease [12]. Therefore, patients present with unspecific heart failure symptoms like reduced exercise capacity, dyspnea, peripheral edema or fatigue. Depending on the organ systems involved, several other symptoms are possible, but there is no specific symptom for amyloidosis [13,14]. However, some findings or comorbidities, respectively, and combinations of different findings are suspicious for amyloidosis and can be summarized as red flags [15]. Cardiac amyloidosis should be suspected in patients with heart failure syndromes and at least one red flag. The diagnostic work-up should therefore include screening for extracardiac findings in addition to the standard procedures that are performed for the evaluation of heart failure symptoms like echocardiography and cardiac biomarkers. Some red flags are typical only for a certain type of amyloidosis, others can occur in every disease form, like hypotension or normal blood pressure if previously hypertensive or worsening tolerance of standard heart failure or anti-hypertensive medications. Pathognomonic extracardiac manifestations of systemic AL amyloidosis are macroglossia [16] and periorbital bleedings [17], but can be observed in only 10–15% of cases. Moreover, AL amyloidosis is a rapid progressive disease, leading to worsening of symptoms in a short period of time within months, compared to ATTR amyloidosis that usually presents as a slowly progressive disease. The medical history of ATTR patients often includes orthopedic conditions [4,18,19,20,21] like bilateral carpal tunnel syndrome, spinal canal stenosis or distal biceps tendon rupture, in many cases years before the onset of heart failure symptoms. Another red flag is the presence of an unexplained polyneuropathy that can occur in ATTRv patients with a mixed phenotype or AL patients. Notably, family history of unexplained cardiomyopathy and/or polyneuropathy might indicate ATTRv, as well as origin from an endemic area. Red flags are summarized in Table 1.

## 3. Laboratory Testing and Tissue Analysis

The first step in the clinical suspicion of amyloidosis should be to determine the free light chains (FLC) in the serum [22], ideally supplemented by immunofixation in serum and urine. If results are abnormal, immediate contact should be made with an amyloidosis center for interdisciplinary planning of further diagnostics, as the diagnosis of AL amyloidosis with rapidly progressive cardiomyopathy appears possible. However, interpretation of the light chain findings can be challenging in some cases: It is known that both impaired renal function and older age can impact physiological light chain levels, which is not taken into account in the common reference ranges. Some authors therefore suggest eGFR- and age-adapted reference ranges. In addition, in the population aged >70 years, monoclonal gammopathy of undetermined significance (MGUS) can be detected in >5% of cases [23]. For further implications of light chain diagnostics, see Section 7.

If cardiac amyloidosis is suspected, the cardiac biomarkers NT-proBNP and troponin are determined, which are highly sensitive but not specific [24,25]. In the case of a diagnosis of amyloidosis, they have a decisive prognostic significance [26,27,28]. Notably, NT-proBNP levels are often out of proportion to the clinical phenotype. In AL amyloidosis, other organ-related parameters such as albuminuria or alkaline phosphatase are also determined due to the possible involvement of kidneys and liver. To ensure diagnosis of AL amyloidosis, usually two tissue samples are required: firstly, the amyloid deposits must be detected using Congo red staining and thereafter amyloid subtyping by immunohistochemistry or mass spectrometry is mandatory. Secondly, the underlying monoclonal B cell disease must be confirmed by bone marrow biopsy. A fluorescence in situ hybridization (FISH) analysis of bone marrow is essential, as the results can have implications for therapy. Congo red staining should also be performed, but sensitivity in bone marrow is only up to 60%, so it is recommended to order in addition a biopsy of abdominal subcutaneous fat tissue, where sensitivity for amyloid detection is >80%. The combined sensitivity of bone marrow and fat tissue biopsy is >90%, so that the affected organs do not need to be biopsied in most cases. In patients diagnosed with ATTR amyloidosis, TTR gene analysis must be performed to avoid missing the hereditary form. A transthyretin (prealbumin) level determination in serum usually has no diagnostic value and is not routinely assessed for treatment monitoring but measurement within clinical trials showed a decrease in TTR levels of up to 80% under therapy with a TTR silencer [29], while under therapy with a TTR stabilizer, an increase in levels could be observed under therapy as an indirect measurement of TTR stabilization [30].

## 4. Echocardiography

Echocardiography (EC) is the most common and best available modality for the diagnostic workup of patients with heart failure symptoms. Several non-specific findings in EC can indicate for cardiac amyloidosis and the combination of different signs can strongly support the diagnosis; however, the final diagnosis of cardiac amyloidosis cannot be confirmed by EC alone and requires additional modalities that are described later on. The most common finding is left ventricular hypertrophy (LVH) or being more precisely: a thickening of the left ventricular wall. The term LVH is somewhat misleading, because amyloid depositions are extracellular, what is not a form of hypertrophy by definition. Especially when no other causes for hypertrophy/thickening like arterial hypertension, hypertrophic cardiomyopathy or aortic stenosis are present, it might be a first indicator for cardiac amyloidosis, whereas a thickness of the interventricular septum >12 mm was defined as a threshold in the past. Notably, a normal thickness does not rule out cardiac amyloidosis, especially in female patients and in early disease stages. Furthermore, a thickening of the right ventricle, the interatrial septum and the valves can be observed frequently [5,31,32]. Another traditional sign is an increased echogenicity of the myocardial walls, known as “granular sparkling”, that can be found in up to 25% of all patients, but can occur in other diseases like myocarditis as well [33]. Pericardial effusion in varying degrees can be observed frequently and is more frequent in AL amyloidosis. The systolic function, measured by left ventricular ejection fraction (LVEF) is usually preserved in early stages cardiac amyloidosis but can decrease during the further course of the disease. Therefore, LVEF cannot serve as a diagnostic criterium but might be helpful for disease staging. Whereas the longitudinal function is typically reduced already in early disease stages. Diastolic dysfunction can be detected in most of the patients with cardiac amyloidosis. The grade of diastolic dysfunction varies, depending on the disease stage and amyloid type. Grade I and II diastolic dysfunction is present in earlier stages, notably, especially in AL even in the absence of significant thickening of the myocardium, whereas grade III diastolic dysfunction with elevated ventricular filling pressures is usually a sign for an advanced disease stage, frequently accompanied with biatrial enlargement [34]. The most powerful technique to differentiate between cardiac amyloidosis and other diseases is speckle-tracking EC, respectively, strain imaging. Reduced longitudinal strain in basal and midventricular segments with preserved longitudinal strain in the apical segments is referred to as “apical sparing”, a pattern that distinguishes amyloidosis from other forms of thickening/hypertrophy with a sensitivity of 93% and specificity of 82% [35]. However, apical sparing can also be observed in patients with storage diseases like Fabry disease or patients with severe chronic kidney disease. Several multiparametric models have been developed to improve sensitivity and specificity for different subgroups, e.g., the AL and IWT scores [36], the ATTR-CM [37] score or the AMYLI score [38].

In summary, EC can provide further support for the suspected diagnosis cardiac amyloidosis and should be the first modality used on the diagnostic pathway. Depending on the disease stage, findings can be different and it is strongly recommended to perform strain imaging due to the high sensitivity and specificity. Further, EC provides prognostic information and enables therapy monitoring.

## 5. Cardiac MRI

Cardiac MRI (CMR) enables high-resolution imaging for the assessment of myocardial function and moreover tissue characterization. Morphological and functional abnormalities in cardiac amyloidosis are similar to EC findings, but different techniques provide additional information about the myocardial tissue like inflammation, infiltration and fibrosis, enabling precise evaluation of a hypertrophic phenotype and classification into hypertrophic cardiomyopathy, hypertensive heart disease, storage diseases, cardiac amyloidosis or other cardiomyopathies. After administration of gadolinium-based contrast agents, late gadolinium enhancement (LGE) can identify areas of the myocardium with increased extracellular volume like fibrosis or amyloid deposition, whereas different LGE patterns indicate different diseases [39]. Cardiac amyloidosis usually shows global subendocardial LGE in early disease stages and global transmural LGE in advanced disease stages. These characteristic LGE pattern not only allows the diagnosis of cardiac amyloidosis but provides prognostic information as well [40]. The determination of T1 relaxation times of the myocardial tissue by the use of T1-mapping techniques can estimate the expansion of the extracellular space due to fibrosis, edema or amyloid deposition despite some technical limitations [41]. Combined with contrast-enhanced T1-mapping, the extracellular volume can be calculated, allowing quantification of amyloid burden [42,43].

**Figure 2:**
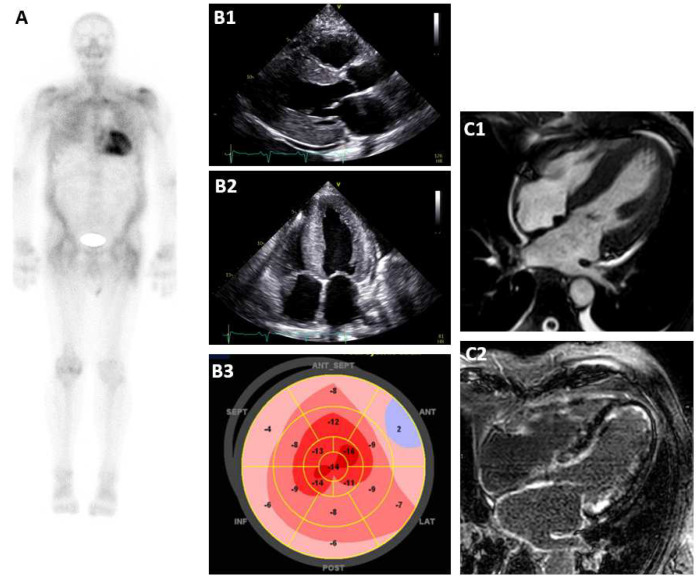
Typical findings in different imaging modalities. (**A**): Bone scintigraphy Perugini score 3, (**B1**): EC parasternal long axis, (**B2**): EC apical 4−chamber−view, (**B3**): apical sparing in strain analysis, (**C1**): CMR 4−chamber−view, and (**C2**): CMR diffuse LGE.

In summary, the diagnostic versatility of CMR allows for excellent differentiation between various forms of cardiac hypertrophy. Furthermore, quantification of amyloid burden is possible with LGE and mapping techniques, making CMR a very effective tool for therapy monitoring, risk stratification and diagnosis of early, even subclinical disease stages. Depending on the local availability, CMR could serve as a first-line diagnostic tool and should be considered in particular for every patient with inconclusive EC results and in cases when other differential diagnoses have an equal probability. Typical findings in different imaging modalities are shown in Figure 2.

## 6. Bone Scintigraphy

It has been known since the early 1990s that ^99m^Technetium-labeled radionuclide bone tracers bind to certain amyloid deposits [44]. The binding mechanism is not fully understood, but it is assumed that microcalcifications in the amyloid deposits and calcium binding sites within the amyloid fibrils play a role in the process. The significantly stronger tracer accumulation in ATTR amyloidosis compared to AL amyloidosis could be explained by different distribution of microcalcifications and calcium binding sites depending on the amyloid fibril subtype [45]. The most commonly used tracers are ^99m^Tc-3,3-diphosphono1,2-propanodicarboxylic acid (^99m^Tc-DPD), ^99m^Tc-hydroxymethylene (^99m^Tc-HMDP) and ^99m^Tc-pyrophosphate (^99m^Tc-PYP), of which the latter is only available in the US. According to Perugini et al. [46], tracer uptake is divided into four grades. Moderate to intense tracer accumulation (grade 2 or 3), combined with unremarkable M-protein diagnostics, achieves a sensitivity and specificity for the diagnosis of ATTR cardiomyopathy of almost 100% [47]. To avoid false-positive findings, it is important to add SPECT (single-photon-emission computed tomography) imaging [48].

## 7. Diagnostic Pathway

**Figure 3:**
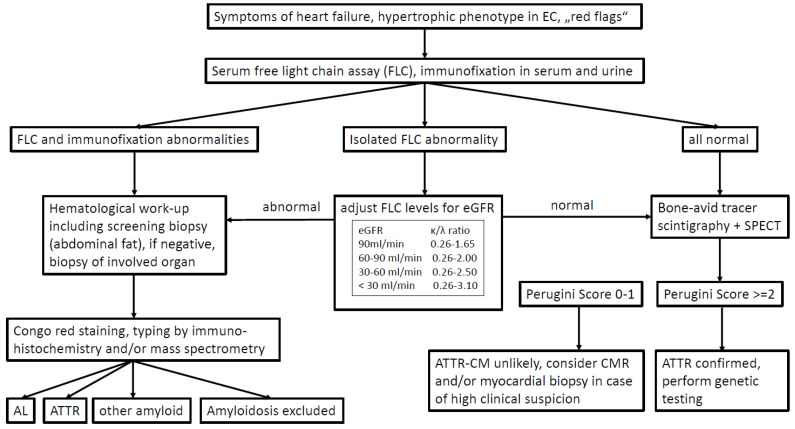
Diagnostic pathway.

In recent years, various cardiologic and hematologic societies have published a number of diagnostic algorithms that differ only in a few details [12,22,49,50,51,52,53]. An algorithm published in 2023 by the European Society of Cardiology (ESC) highlights the importance of early free light chain (FLC) diagnostics as described above (Figure 3). If cardiac amyloidosis is clinically suspected, which is usually based on heart failure symptoms and typical abnormalities in echo or cardiac MRI, the next step should be to determine FLC in serum and immunofixation in serum and urine (spontaneous urine is sufficient). In the case of abnormal light chain levels, it is important to note whether one light chain is significantly elevated and immunofixation actually indicates a monoclonal gammopathy or whether light chain levels are only slightly abnormal, immunofixation is negative and chronic kidney disease is present. In the latter case, the abnormal light chains can be explained by impaired kidney function as eGFR-adapted FLC ratio reference ranges are suggested for patients with chronic kidney disease (see Figure 3). In this situation, the subsequent bone scintigraphy with grade 2/3 cardiac tracer uptake can lead to the non-biopsy diagnosis of cardiac ATTR amyloidosis. A TTR gene mutation analysis must then be added to discriminate between hereditary and wild-type disease. If a monoclonal gammopathy was detected in the light chain diagnostics, the further diagnostic steps depend on which amyloidosis appears more likely in the context of the findings (age, evidence of extracardiac manifestations, level of monoclonal free light chains in the serum).

- If ATTR amyloidosis is suspected despite monoclonal gammopathy, this must be confirmed using a tissue sample, regardless of a positive bone scintigraphy, and since the sensitivity for ATTR amyloidosis in peripheral screening biopsies (fat, GI tract) is low, usually an endomyocardial biopsy is required.
- If AL amyloidosis is suspected, bone scintigraphy can be postponed and a biopsy should be performed directly, preferably from the abdominal subcutaneous fat tissue with a sensitivity of >80% [54]. If this screening biopsy is negative, a biopsy of the affected organ must be performed.

## 8. Genetic Testing

Testing for amyloidogenic mutations in the *TTR* gene should be performed in all patients with proven ATTR amyloidosis. The probability of a hereditary form of the disease decreases substantially with increasing age. However, some ATTRv patients can develop symptoms quite late, thus no definitive cut-off age for genetic testing can be defined. The different mutations cause different patterns of disease onset, organ involvement and symptoms and the penetrance may vary by pathogenic variant, geographic region and ethnic group. The most common mutations are listed in Table 2. If a TTR variant can be detected, neurological examination should be performed to screen for polyneuropathy, especially because additional treatment options, like silencer therapies, are available for ATTRv with polyneuropathy. Moreover, genetic counselling should be offered to relatives, the transmission is autosomal dominant. It should also be noted that an overlap of different cardiomyopathies with a similar phenotype can occur in some individuals and a genetic panel diagnostic for hypertrophic cardiomyopathy (HCM) should be considered in selected patients, especially if features of both, amyloidosis and HCM, are present in cardiac imaging.

## 9. Staging

Biomarker-based staging systems were implemented in the last years for AL and ATTR amyloidosis. The most common systems are listed in Table 3 (AL) and Table 4 (ATTR).

## 10. Early Diagnosis of Systemic Amyloidosis/Amyloidosis as an Incidental Finding

Due to the correlation between the extent of organ involvement and the patient’s prognosis, it is crucial to diagnose amyloidosis as early as possible. Ideally, this is performed at the asymptomatic stage, but this is a major challenge because the clinical and imaging findings are non-specific in the early stages of the disease. Hemato-oncologists have the opportunity to obtain early indications of the possible development of AL amyloidosis by regularly determining cardiac and renal biomarkers (NT-proBNP, albuminuria) when observing patients with monoclonal gammopathies, especially those with supposedly lacking clinical significance. With regard to ATTR amyloidosis, early histopathological evidence of ATTR amyloid deposits in tissue samples from carpal tunnel surgery is increasingly being seen. Stepwise cardiac diagnostics should be performed to determine whether the heart is already involved (NT-proBNP determination and, if level is abnormal, bone scintigraphy and echocardiography or cardiac MRI). It should be emphasized that tracer accumulation in bone scintigraphy alone is not equivalent with manifest amyloidosis-associated cardiomyopathy. To make this diagnosis, clinical, laboratory and other imaging information should be considered together, as described above.

Atrial fibrillation is very common in patients with amyloidosis due to amyloid deposition in the atria and patients may be treated for atrial fibrillation or stroke before the diagnosis of cardiac amyloidosis is made. Therefore, screening for amyloidosis should be considered in these patient cohorts, especially if patients have recurrence of atrial fibrillation after ablation, as a recent study indicates that patients with amyloidosis have a high scar burden and therefore an elevated risk for recurrence [57]. Moreover, the risk for stroke events is elevated for amyloidosis patients, thus anticoagulation is indicated independent from the CHA2DS2-VASc score and should be considered even after left appendage occlusion [58].

## 11. Future Perspectives

**Figure 4:**
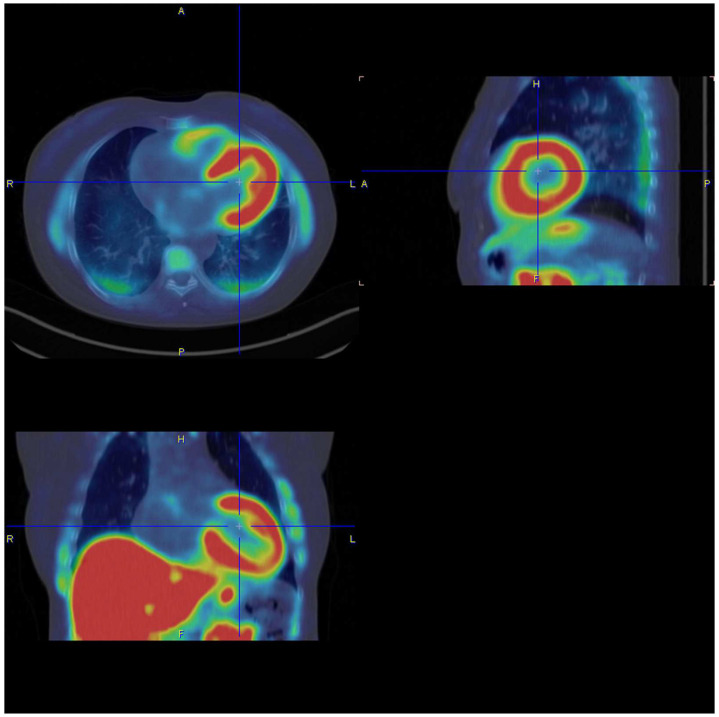
18F-florbetaben-PET/CT scan of a patient with cardiac AL amyloidosis (image by courtesy of [60]).

In the future, it is hoped that the use of artificial intelligence, e.g., in ECG diagnostics, cardiac imaging or tissue analyses, will further improve early diagnosis of amyloidosis [59]. Moreover, new laboratory tests to determine the amyloidogenicity of monoclonal free light chains in serum are tested to identify patients at high risk of developing amyloidosis who still have normal organ function parameters (AmylLite ^TM^). Regarding imaging techniques, several studies are currently being conducted to test different PET tracer that can detect different amyloidosis subtypes (e.g., [18F]-florbetaben, ^124^I-evuzamitide, Figure 4) and possibly may allow discrimination between AL and ATTR amyloidosis as well as quantifying and monitoring of amyloid load [60]. Thus, novel PET tracers could enable a more accurate and easier diagnostic of cardiac amyloidosis as well as monitoring of therapy response. Nonetheless, more investigation is required for the advancement and verification of PET-based imaging.

## 12. Summary

To diagnose cardiac amyloidosis, a combination of clinical features, imaging and biomarkers is required. The correct interpretation of all items is crucial to avoid misdiagnosis. Therefore, a multidisciplinary approach among cardiology, hematology and nuclear medicine is essential.

## Figures and Tables

**Table 1:** Red flags for cardiac amyloidosis.

	Red Flag	Amyloid Type
Anamnesis	Rapid progressive heart failure symptoms (months)	AL
	Disappearance of arterial hypertension	All
	Unexplained polyneuropathy and/or cardiomyopathy in the family history	ATTRv
	Origin from an endemic area (e.g., Portugal, Sweden, Africa)	ATTRv
	History of (bilateral) carpal tunnel syndrome, spinal canal stenosis, distal biceps tendon rupture	ATTRwt, ATTRv
Clinical examination	Macroglossia	AL
	Periorbital bleedings	AL
	Ascending, distal polyneuropathy, autonomic dysfunction	AL and ATTRv
Biomarker	High levels of NT-proBNP, discordant to NYHA class	All
	Albuminuria	AL
	Alkaline phosphatase elevation	AL
ECG	Low voltage, discordance between QRS voltage on ECG and wall thickness	All
	Pseudo-infarct	All
Echocardiography	Pericardial effusion	AL, (ATTR)
	Reduced longitudinal function	All
	Reduced global longitudinal strain (GLS)	All
	Apical sparing	All
Cardiac MRI	Diffuse LGE patterns	All
	Elevated T1 relaxation times	All
	Elevated extracellular volume (ECV)	All

**Table 2:** Common TTR variants.

Variant	Phenotype	Endemic Regions
*p.Val50Met (Val30Met)*	Early onset: neuropathic Late onset: mixed	Portugal, Sweden, Japan, Brazil
*p.Thr80Ala(Thr60Ala)*	Mixed	England, Ireland, Scotland, Greece
*p.Val142Ile (Val122Ile)*	Cardiac	Africa, African Americans
*p.Val40Ile (Val20Ile)*	Cardiac	Germany
*p.Ile88Leu (Ile68Leu)*	Cardiac	Italy, Bulgaria, Germany
*p.Phe84Leu (Phe64Leu)*	Mixed	Italy
*p.Ser70Arg (Ser50Arg)*	Neuropathic	Mexico, Italy, France, Japan

**Table 3:** European Mayo Staging System for AL amyloidosis [55,56].

Biomarker	Stadium I	Stadium II	Stadium IIIa	Stadium IIIb
cTnT [ng/mL]	<0.035	>0.035	>0.035	>0.035
	and	or	and	and
NT-proBNP (pg/mL)	<332	>332	>332 (but ≤8500)	>8500
Median OS (Mo.)	130	54	24	4

**Table 4:** Staging systems for ATTR amyloidosis.

Staging System	Parameters	Median Survival Stage 1 (0 Parameter)	Median Survival Stage 2 (1 Parameter)	Median Survival Stage 3 (2 Parameters)
Mayo (ATTRwt) [27]	Troponin-T > 0.05 ng/mL NT-proBNP > 3000 pg/mL	66 months	40 months	20 months
NAC (ATTRwt, ATTRv) [26]	eGFR < 45 mL/min NT-proBNP > 3000 pg/mL	69 months	47 months	24 months

## Data Availability

No new data were created or analyzed in this study.
